# Antimicrobial Stewardship Program Implementation in a Saudi Medical City: An Exploratory Case Study

**DOI:** 10.3390/antibiotics10030280

**Published:** 2021-03-09

**Authors:** Saleh Alghamdi, Ilhem Berrou, Eshtyag Bajnaid, Zoe Aslanpour, Abdul Haseeb, Mohamed Anwar Hammad, Nada Shebl

**Affiliations:** 1 Department of Clinical Pharmacy, Faculty of Clinical Pharmacy, Albaha University, Albaha 65779-77388, Saudi Arabia; saleh.alghamdi@bu.edu.sa (S.A.); m.anwar@bu.edu.sa (M.A.H.); 2 Faculty of Health & Applied Sciences, University of the West of England, Staple Hill, Bristol BS16 1DD, UK; 3 Department of Clinical Pharmacy, Pharmaceutical Services Administration, King Abdullah Medical City, Makkah 11176, Saudi Arabia; bajnaid.e@kamc.med.sa; 4 Department of Clinical and Pharmaceutical Sciences, School of Life and Medical Sciences, University of Hertfordshire, Hatfield AL10 9AB, UK; z.aslanpour@herts.ac.uk (Z.A.); n.a.shebl@herts.ac.uk (N.S.); 5 Department of Clinical Pharmacy, College of Pharmacy, Umm Al Qura University, Makkah 77207, Saudi Arabia; amhaseeb@uqu.edu.sa

**Keywords:** antimicrobial stewardship programs, hospitals, multi-drug resistance

## Abstract

Antimicrobial stewardship programs (ASPs) in hospitals have long been shown to improve antimicrobials’ use and reduce the rates of antimicrobial resistance. However, their implementation in hospitals, especially in developing countries such as Saudi Arabia, remains low. One of the main barriers to implementation is the lack of knowledge of how to implement them. This study aims to explore how an antimicrobial stewardship programme was implemented in a Saudi hospital, the challenges faced and how they were overcome, and the program outcomes. A key stakeholder case study design was used, involving in-depth semi-structured interviews with the core members of the ASP team and analysis of 35 ASP hospital documents. ASP implementation followed a top-down approach and involved an initial preparatory phase and an implementation phase, requiring substantial infectious diseases and clinical pharmacy input throughout. Top management support was key to the successful implementation. ASP implementation reduced rates of multi-drug resistance and prescription of broad-spectrum antimicrobials. The implementation of ASPs in hospital is administrator rather than clinician driven. Outsourcing expertise and resources may help hospitals address the initial implementation challenges.

## 1. Introduction

Antimicrobial stewardship programs (ASP) in hospital are interventions to reduce risks of antimicrobial treatment failures, adverse events, hospital acquired infections, rates of antimicrobial resistance and costs associated with antimicrobial prescriptions and prolonged length of stay in hospital [1]. These programs focus on optimizing the choice, dosing and route of administration of antimicrobials, monitoring their prescription and resistance patterns, and continuous provision of education and feedback to prescribers.

The programs were first implemented in the USA in the late 1990s, and started to gain popularity in Europe soon after that [2]. In an international survey carried out in 2015 by the ESCMID Study Group for Antimicrobial Policies (ESGAP) and ISC Group on Antimicrobial Stewardship, 52% of the countries had a national antimicrobial stewardship plan, 58% had ASPs in hospitals and 4% of the countries were planning antimicrobial stewardship strategies [3]. The Netherlands, France [4] and England [5] reported high rates of ASP implementation in hospitals. In the USA, a recent study suggests that 51% of the leading hospitals in the country have an active ASP. Around 59% of these hospitals had ASP for more than 5 years [6]. However, the status of ASP implementation in hospitals in developing countries is less clear. Various reports point to low levels of implementation [7,8,9] for various reasons including: a lack of diagnostic testing and antimicrobials, sub-optimal infection prevention and control practices and the prevalent inappropriate prescribing practices [10,11]. Resistant microorganisms may spread from one person to another, one health care facility to another and one country to another [12]. Tackling antimicrobial resistance in developing countries is critical to reduce its burden in those countries, and to strengthen the global effort to contain this threat.

Like in many other developing countries, Saudi hospitals report low levels of ASP implementation, mainly in tertiary hospitals, despite having a national antimicrobial stewardship plan to implement ASPs in hospitals [13]. This is in contrast to reports of high prevalence of antimicrobial resistance and the emergence of new and rare multi-drug resistant strains [14]. Worryingly, these pathogens can spread globally given that Saudi Arabia is a popular destination for millions of international travelers annually for pilgrimage (Hajj). 

We recently reported that one of the factors behind the lagging implementation of ASPs in Saudi hospitals is the lack of “know how” to implement them [13]. ASPs are novel to Saudi hospitals, and their implementation would require a significant change to routine practice, where antimicrobials are heavily prescribed, often inappropriately [15,16]. A recent guide by Mendelson et al. details a generic protocol for the implementation and/ or optimization of ASP in hospitals in both developing and developed countries’ contexts [17]. However, three issues may be considered. First, the protocol may provide a useful outline of the resources needed and what the programs usually entail, but it may not be sufficiently practical, and may not highlight how implementation challenges can be practically overcome. Second, although the protocol recognises the resource limitations in developing countries, it does not differentiate between implementation in developing vs. developed contexts. Third, the protocol is clinician-oriented; however, in various developing countries, such as Saudi Arabia, it is hospital administrators, not clinicians, who often make decisions about policy implementation [15]. This study aims to explore how an antimicrobial stewardship programme was implemented in a Saudi hospital, the challenges faced and how they were overcome, and the program outcomes. Findings would improve the current knowledge of ASP implementation in developing countries, and provide hospitals in the region with a practical guide to implement these programs.

## 2. Results

A number of areas emerged as key themes from the interviews: motives for ASP adoption, development and implementation of ASP, implementation challenges, and outcomes. Where useful, we included quotes from the participants to illustrate key points. 

### 2.1. Motives for ASP Adoption in the Medical City

The decision to adopt and implement an antimicrobial stewardship programme was initially made by the chief executive officer (CEO) of the medical city in 2015. 

“Leadership support is the biggest thing we have here (at the hospital). It was all from the leadership to begin with. After 2–3 weeks from my appointment, the CEO came to me and said I want to start this (antimicrobial stewardship) programme, and it has to be up and running…”

Various factors (stated in no particular order of importance) seem to have influenced this decision. First, the CEO’s surgical background heightened the need to improve infection prevention and control, and reduce the prevalence of multi-drug resistant strains in the facility. The CEO’s training in an international hospital with a longstanding experience of ASPs influenced their conviction of the benefits of ASPs in the organisation. Second, the CEO perceived the medical city, one of the largest and most highly specialist tertiary centres in the country, to be a pioneer, and an exemplary model for other hospitals in the region to follow suite in relation to implementing ASPs. Third, the special context of the medical city, being a referral facility from other hospitals in the region, means that they often admit patients with complex care needs, who would have spent months in the referring hospitals. These patients tend to be colonized with multi–drug resistant strains, which are then transferred to the medical city. Fourth, the medical city is home to advanced neurosurgery and oncology centres, whose patients are immunocompromised and vulnerable to severe outcomes of multi-drug resistant infections. This context necessitated the adoption and implementation of interventions, such as ASPs, that can reduce the prevalence of multi–drug resistance and optimise the use of antimicrobials.

From the infectious diseases’ consultant, clinical microbiologist, the lead antimicrobial pharmacist and infection control consultant’s perspectives, the strongest motive to adopt and implement ASPs was the high prevalence of multi-drug resistant (MDR) microorganisms, particularly among Gram–negative bacteria. This was fueled by the high and inappropriate use of antimicrobials. The high prevalence of MDR organisms may have contributed to increased mortality rates, failure of surgical procedures, and may have compromised the safety of immunocompromised patients receiving specialist oncology, cardiac and neurosurgery services. The hospital was also planning to set up a transplant centre and was desperate to reduce the rates of multi-drug resistance. 

“They were prescribing very expensive and inappropriate antibiotics and antifungals …ICU patients were mostly on 3, 4, or 5 antibiotics. It was not justifiable at all… We needed to start a strong antimicrobial stewardship programme for the patient’s sake, and also to educate prescribers”.

“We were having outbreaks of multi-drug resistant organisms and there was overuse of antibiotics. We had a major issue with drug resistant *Acinetobacter baumannii* which was sensitive to colistin, and then it became resistant to colistin. So, it became pan-drug resistant *Acinetobacter baumannii*. The infection control experts (in the hospital) said that this was because of the overuse of carbapenems in the ICU, and then the overuse of even colistin in the ICU”.

“We have the oncology centre and we wanted to start the transplant centre, and of course the immunocompromised patients needed to have much fewer resistant microorganisms”.

It is noteworthy that the MOH antimicrobial stewardship plan does not appear to be a motive for ASP adoption and implementation despite the medical city being a “flagship” MOH organisation. 

### 2.2. Development and Implementation of the Antimicrobial Stewardship Programme

#### Phases of ASP Implementation

**Figure 1:**
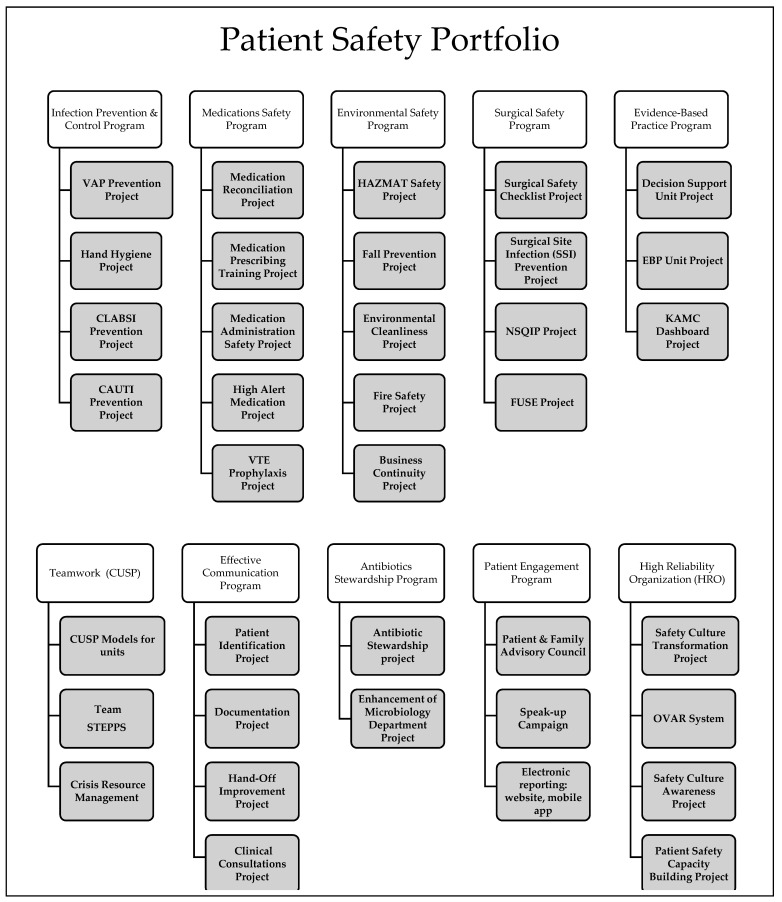
Patient safety portfolio of programs.

The ASP is part of the hospital’s patient safety portfolio (PSP) of 10 strategic patient safety programs to address patient safety issues within the hospital and to improve the quality and safety of care. These are shown in Figure 1.

There were two phases of ASP implementation in the medical city. 

Phase 1

The ASP aims to improve antimicrobial prescribing practices, reduce the high prevalence of multi–drug resistance in the hospital and the high costs associated with antimicrobials’ prescribing.

Initially, the ASP programme was suggested to be part of the medication safety program, but the CEO insisted that the ASP programme should be an independent, stand-alone programme with its own key performance indicators.

“When we started discussing antimicrobial stewardship, the idea was that it should be part of the medications (safety) programme, but I said no, it needs to be done independently, it needs to stand out, to be very obvious and very evident”

The hospital had no expert staff “in house” to set up and implement the ASP, so it outsourced a locum infectious disease pharmacist, who trained in a hospital in the USA. The locum ID pharmacist reviewed current practices and the potential implementation barriers, and provided practical guidelines on how these could be overcome. The hospital then appointed an antimicrobial lead pharmacist, who worked with the locum ID pharmacist, and the ID consultant to draft the hospital ASP policy (based on the Infectious Diseases Society of America (IDSA) ASP guidelines) [1,18], the formulary restriction list (based on local antibiogram and MDROs surveillance reports), and set up the ASP team.

“we started with first thing formulary restriction based on the MDR (multi-drug resistance) pattern at the hospital. What are the medicines that we need to restrict firstWe started with broad spectrum antibiotics…carbapenems, meropenem, imipenem, colistin…and then with the antifungals voriconazole, posaconazole…then we included caspofungin, micafungin and anidulafungin since they have a high cost…”

The core members of the ASP team at the medical city were:

- The ID consultant as the director of the ASP program and their ID team;
- The antimicrobial lead pharmacist as the manager of the ASP program and the clinical pharmacists’ team;
- Clinical pharmacists;
- Consultant clinical microbiologist and their team;
- The infection control consultant and their team.

**Figure 2:**
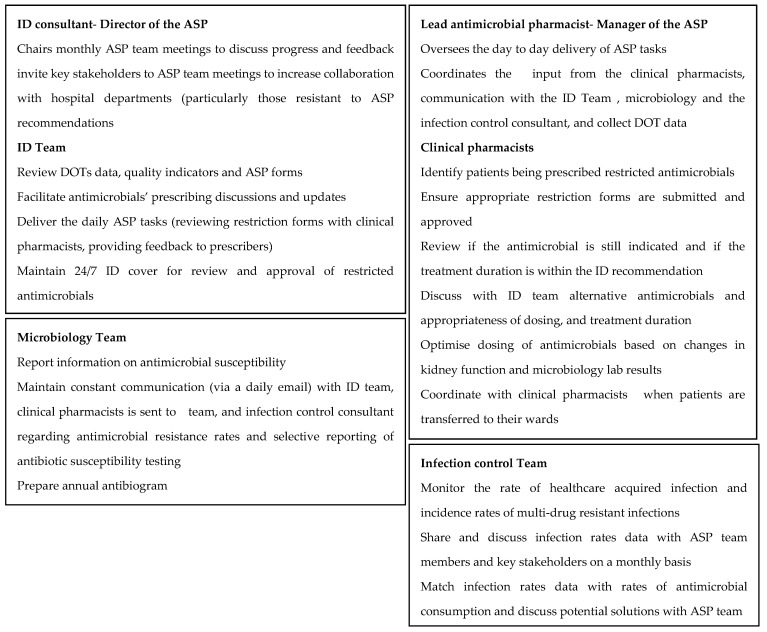
Roles and responsibilities of core members of the ASP team. ASP (antimicrobial stewardship programme); ID (Infectious Diseases); DOT (Days of Therapy).

The roles and responsibilities of each team member are presented in Figure 2. The ASP team maintained direct (and frequent) communication with key stakeholders such as the heads of ICU, hematology and oncology departments (where antimicrobials’ use and resistance were high), and the head nurse (whose team is directly affected by the change in how antimicrobials will be obtained and administered). Those key stakeholders were often invited to take part in ASP team meetings, and become members of the bigger ASP team.

The team developed an antimicrobial restriction form and discussed it with hospital physicians, and clinical and hospital pharmacists. The form was introduced on a trial basis, to get physicians used to filling in the form and discussing prescription decisions with the ID consultant. It was then updated based on prescribers’ and pharmacists’ feedback, before approval by the medical records committee. A copy of the antimicrobial restriction form and the associated workflow is included in Supplementary File S1.

The remaining steps of the implementation plan were then agreed; these included: 

Raising awareness of antimicrobial resistance and the outcomes of inappropriate antimicrobials’ use through a hospital-wide health promotion event lasting for a week and coinciding with the World Antimicrobial Awareness Week (18–24 November 2015). 

“everyday was targeted to specific people or specific departments. We published a newsletter about antimicrobial stewardship and we had small antimicrobial stewardship contests”.

Antimicrobials prescribing training sessions for physicians:

“We let people get used to filling the forms, and know which antibiotics are restricted. Before there was resistance, and now everyone knew that it is just a matter of telling, understanding, and providing feedback…it will be applied and implemented as restricted antibiotics will not be approved unless it is necessary. If it (restricted antibiotic) is prescribed, it will not be approved by the ID team (unless the form is filled in)”

Training members of the ID team and the clinical pharmacists on how to carry out the daily ASP tasks once the programme goes live at beginning of January 2016. 

Phase 2

The two core strategies (restriction and preauthorization, and prospective audit with intervention and feedback) of ASPs were implemented. In January 2016, the plan was to introduce the restriction form into hospital departments with the least prescriptions of broad-spectrum antimicrobials (neuroscience and surgery) to identify potential implementation issues, and manage the workload of the small ID team at the time. However, the hospital decided to roll out the form into all the remaining departments including ICU, hematology and oncology because of the recruitment of more ID clinicians, which expanded the capacity of the ID team to review and authorize physicians’ requests of restricted antimicrobials. Furthermore, the ID team provided 24 h, 7 days a week “on call” cover dedicated to reviewing and authorising (or not) restriction forms. 

“Each unit is covered by ID (Infectious disease team) for these forms. They meet every morning. They meet with the clinical pharmacist in that unit. They will also review all the (restriction) forms, and if it is justified they will approve it. If they are not satisfied, they look into the patient’s file, and then discuss with the prescribers. The discussion with the prescriber is mainly because we want the education to play a role (in the programme)”.

In addition to antimicrobials’ restriction, the ASP team conducted regular auditing of antimicrobials’ prescribing, communicated rates of antimicrobials’ prescribing to relevant heads of departments, and provided feedback to prescribers. Prospective audit and feedback were also carried out by the pharmacy team to ensure adherence to antimicrobials’ guidelines and optimise antimicrobials’ dosing before input from the ID team; the infectious disease physicians and the clinical pharmacists maintained regular contact with the medical team regarding patients on antibiotics. The clinical pharmacists were responsible for documenting the daily follow ups or recommendations on the patients’ files. They would document their input under the title “ASP Pharmacy Assessment”.

The microbiology team started selective reporting of antibiotic susceptibility testing to reduce physicians’ prescribing of restricted antimicrobials such as meropenem and imipenem:

“We follow cascade reporting…microbiology (started) to minimise the disclosure of all susceptibility reporting”.

The ASP team continued to meet monthly to review rates of antimicrobial resistance, DOT (days of therapy) data and the list of restricted antimicrobials. Feedback from physicians and heads of department regarding antimicrobials’ prescribing needs was discussed and addressed. Examples include drafting guidelines on the prescribing of oral fosfomycin for UTIs, surgical prophylaxis, granulocytopenia and vancomycin dosing.

“Most of our physicians do not use Fosfomycin because they do not believe that oral medication can be used against multi-drug resistant organisms like ESPL (Extended-Spectrum Beta-Lactamases) or CRB (Chlorine-Resistant Bacteria)…we have a lot of patients now deescalating to fosfomycin”

**Figure 3:**
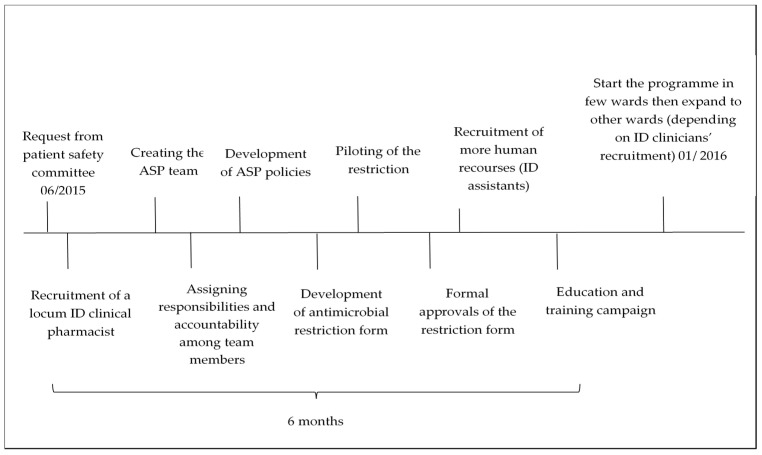
The timeline for implementing the ASP in the medical city.

Education efforts were ongoing throughout the second phase of implementation. Prescribers’ education has been a fundamental component for ASP success at the medical city. Figure 3 shows the timeline for implementing the ASP in the medical city.

### 2.3. ASP Implementation Challenges

Various factors affected the implementation of ASP in the medical city.

#### 2.3.1. Shortage of ASP Staff

During the initial phase, the availability of only one ID consultant, and no other clinician with ID expertise forced the ASP team to optimise ID input, and resulted in an initial “short” list of restricted antimicrobials requiring approval from the ID consultant.

“We started with one ID consultant, we did not have anyone else. That’s why we needed as much concise shortlist as we can”.

During phase 2, the hospital recruited more ID clinicians, resulting in rolling out the ASP across all hospital departments. The shortage of ID physicians has been attributed to the lack of infectious diseases training programs for physicians in the Middle East and most Asian countries. 

Shortage of microbiology personnel and facilities at the medical city also challenged the implementation of the programme. The medical city had only one consultant clinical microbiologist, heading a team of microbiology technicians. Supply issues of laboratory consumables and equipment, due to financial pressures, placed constraints on how quickly susceptibility tests were reported:

“we do not have items coming regularly. Sometimes we even run out of gram stain reagents and we need to borrow them from other hospitals…we would like to have some key equipment like MALDI/TOF (matrix-assisted laser desorption ionization time-of-flight mass spectrometry)... we all know there are financial constraints…so we have supply issues, space issues and financial issues”.

#### 2.3.2. Incompatible IT Systems

One of the biggest challenges to ASP implementation at the medical city, as reported by the participants, was the incompatibility of the electronic medical system in the hospital with the requirements of the ASP, so several data had to be generated manually (antimicrobials’ costs data, DOT data and data for the hospital antibiogram). Furthermore, the restriction forms could not be submitted electronically, they had to be filled in manually, and hard copies had to be collected by pharmacists, and reviewed and authorised by ID physicians:

“we have some constraints in our hospital information system…we are trying to work around that”;

“The health information system at the hospital is not supportive enough, to get accurate data, as we collect data manually (in all departments), which consumes time and manpower”.

Physicians’ resistance to ASP formulary restrictions and policies

The physicians were initially resistant to formulary restrictions and the need to obtain ID approval for the prescription of restricted antimicrobials for numerous reasons:

1. Physicians’ worry about complications given that their patients tend to be complex, immunocomprosied and systemically unwell.

“At the beginning we faced resistance, especially in the critical areas in haematology and oncology because they (physicians) say our patients are sick”.

2. During phase 1, the resistance appears to be mainly towards ID clinicians’ involvement. The physicians at the hospital were routinely making antimicrobial prescription decisions without seeking input from the only ID consultant; who would not have been able to provide input to all departments. During phase 2, after the ID consultant, and later the ID team, became more involved in decisions about antimicrobial prescriptions, the physicians started to seek more consultations and input from the ID team:

“Now the doctors trust more the ID (infectious diseases) team with their consultations. I think also since the ID team started to be more involved with the restriction and talking to convince the doctors that this needs de-escalation…”.

3. Resistance to change: Before the implementation of the ASP, the physicians routinely prescribed antimicrobials empirically and prescribed more than one broad-spectrum antimicrobial, without much reliance on susceptibility reporting. The restriction of prescribing options, and the need to rely on susceptibility reporting and ID approval, was a significant change to their routine practice.

“People tend to treat empirically instead of trying to diagnose”.

### 2.4. Critical Factors for the Sucessful Implementaion of ASP in the Medical City

A number of factors have been identified as key to the implementation of the ASP in the hospital. 

#### 2.4.1. Top Management Support

The decision to adopt and implement ASP in the medical city was made by the CEO. Top management support ensured dedicated financial resources for ID clinicians’ and locum ASP pharmacist recruitment. Furthermore, senior managers instructed the hospital departments to engage with the ASP team’s educational events and process changes. This support was provided throughout the implementation phases, and was perceived by the participants to be a key determinant of the successful implementation of the ASP:

“There is a lot of commitment from the administration and the leadership”;

“Leadership support is the biggest thing we have here”.

#### 2.4.2. Project Management Training

When the PSP programmes and projects were outlined, the hospital administration provided project management training (outsourced) to clinicians involved in these projects including the ASP project. This entailed training on how to write policies and project proposals, identify project outcomes, carrying out relevant data analysis, and strategies to influence behavior change.

#### 2.4.3. A Dedicated ASP Team

A key facilitator to ASP implementation was setting up a dedicated ASP team. This meant that there was a specific person (or group) that maintained open, constant communication with the hospital staff regarding ASP, and was always available for assistance with the implementation of the program. The group members had clear roles and responsibilities and communicated frequently to track the progress of the implementation plan and evaluate its outcomes.

#### 2.4.4. Increased ID Clinicians’ Involvement in the Prescribing and Monitoring of Antimicrobials

The implementation of ASP in the hospital aimed to change physicians’ antimicrobial prescription behaviors, through the restriction of certain antimicrobials, the prescribing of which would require the input of an ID clinician. The ID team ensured that an ID clinician was available over 24 h, 7 days a week to review restriction forms, authorise requests if appropriate and suggest alternative antimicrobials if needed. This increased provision facilitated physicians’ cooperation and reduced their resistance to the process changes: 

”even weekends they (ID team) come to just sign and review the forms, and there is always an ID on call for antimicrobial stewardship beside the ID on call”.

The ID team also delegated antimicrobials’ dose optimisation to the pharmacy team in recognition of clinical pharmacists’ skills and expertise, and to manage the workload associated with the increased cover:

“The ID consultant sent a memo to the whole hospital that the ID consultant, ID doctors and hospital physicians will recommend the regimen, and the dosing will be the responsibility of the pharmacist. That was a huge thing, and everyone was following this recommendation”.

**Figure 4:**
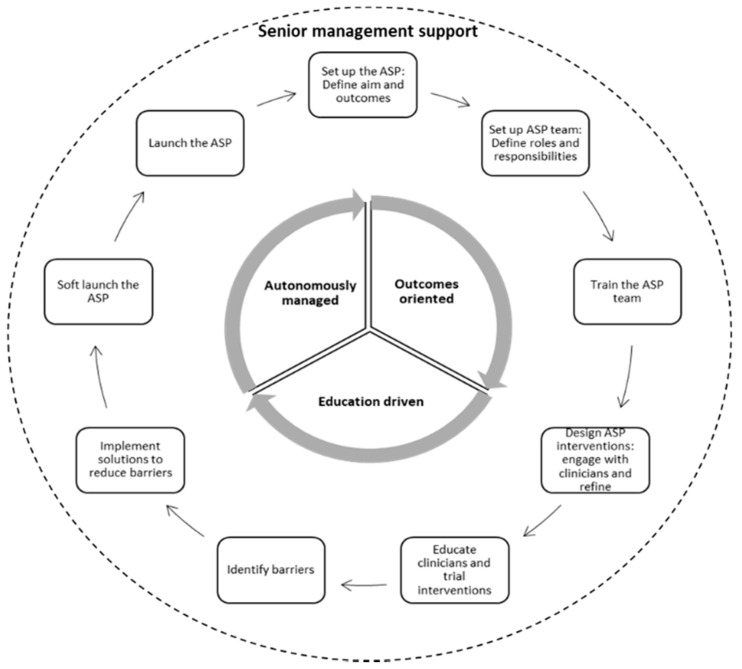
ASP implementation process model.

Figure 4 summarizes the nine essential steps for ASP implementation in hospital, fostered by strong senior management support and governed by key implementation strategies. Hospitals in the region should first start by setting up the ASP program as a stand-alone program with defined aims and outcome measures. Then, an ASP team needs to be set up with clear roles and responsibilities, especially in relation to the day-to-day management of the implementation and oversight of the program. The ASP team would need to be trained on how to manage the project, carry out the implementation steps, evaluate interventions and modify the implementation plans based on feedback from users. Relevant ASP interventions would then have to be designed and refined based on clinicians and administrators’ feedback, followed by education campaigns and training sessions with the clinicians to improve their engagement and reduce their resistance. Throughout the constant engagement with clinicians and hospital administrators, barriers to implementation need to be identified and addressed prior to implementation. The ASP program can be piloted in departments with the least antimicrobial prescribing and resistance issues, before launching it throughout the remaining departments. This is a cyclical process, and sustaining successful outcomes and good practice may require refining the ASP aims and outcomes, adding more members to the ASP team, further education and training and ongoing exploration and identification of barriers to adherence to ASP policies and procedures. Senior management support is paramount throughout the implementation process. The ASP team should have autonomy in managing and refining the ASP, should focus on achieving the defined ASP outcomes, and employ an education approach to help clinicians adhere to the restrictive requirements of the program.

### 2.5. Outcomes of ASP implementation

The ASP implementation outcomes set out by the ASP team were: reduction of antimicrobial resistance rates, rates of multi-drug resistant microorganisms, antimicrobials’ usage (DOTs) and costs (we were not able to obtain data for all the outcomess). These were used as key performance indicators (KPIs) and were regularly monitored by the ASP committee.

Following the implementation of the ASP in 2016, the hospital achieved a reduction in resistance rates (Table 1) and antimicrobials’ usage (Table 2). The hospital either sustained or slightly increased levels of the susceptibility of microorganisms to antimicrobials throughout 2017–2019. However, antimicrobial usage seems to have increased over the years. DOT data of 2019 shows a particularly marked increase compared to data from 2018 (data shown in Tables S1 and S2 of Supplementary File S2) This increase has been attributed to a number of reasons (suggested through personal communication) including: setting up a solid organ transplant center in 2018 and the associated increase in patient numbers and prescribing antimicrobials, an incremental 20% increase in patients’ numbers annually and increased numbers of extended-spectrum β-lactamase (ESBL) isolates due to the restriction of prescribing board spectrum antimicrobials, which led to increased use of piperacillin/tazobactam, cefepime and quinolones. Furthermore, Meropenem’s use doubled, and micafungin’s use tripled from 2018 to 2019. In 2019, the hospital decided to reduce the use of imipenem and limit its use to resistant *Enterococcus facium*, due to cost. The alternative, Meropenen, was more cost-effective, and was being prescribed instead. Similarly, Micafungin was being prescribed as an alternative to the more expensive antifungals caspofungin and anidulafungin in late 2018. Later in 2019, anidulafungin was re-added to the formulary for limited patients with liver impairment.

In late 2020, we sought updates on the status and outcomes of the ASP in the hospital. Supplementary File S3 includes a summary of personal communications with the hospital’ s ASP team.

## 3. Discussion

Top management initiation and support of ASP implementation in the hospital, combined with a team approach to planning, implementing and monitoring of the ASP has led to the successful implementation of the program in the hospital. The implementation challenges reported in this study have been reported nationally in Saudi Arabia [13], but this hospital demonstrates that implementation remains possible if key players (top management and ASP team members) work together, effectively, to address those challenges. 

In this case study, we demonstrate how managerial and clinical interests can be aligned to reduce antimicrobial resistance rates, and pioneer the implementation of ASPs to enhance the hospital’s reputation. Alignment of managerial and clinical interests has been shown to be a key determinant of the success of quality improvement interventions [19]. Furthermore, in contrast to a typical top-down order delegation approach, the CEO empowered the members of the ASP team to take over the responsibility of ASP, monitor its key performance indicators, and make autonomous decisions. This empowering leadership strategy was key to the successful implementation of ASP in our study, and was also reported in Steinmann et al. study [20].

The initial lack of “know how” to implement ASPs and the national shortage of ASP team members have widely been reported to hinder ASP implementation [15,21]. However, top management support ensured the allocation of the necessary funds and resources, and outsourcing expert staff to help hospital staff implement ASPs. Other reported examples also include outsourcing laboratory services [22] or pharmacy services [23]. ASP implementation in the hospital could only occur once more ID clinicians were recruited. Other hospitals may not be able follow example given the national shortage of these specialists. To overcome the shortage of ID clinicians, hospitals in South Africa implemented an alternative pharmacist-led ASP [24], highlighting the need to adapt ASP programs and interventions to maximise the use of scarce resources. We also suggested in [13] that regional/local ASP hubs can be set up so that ID and clinical pharmacists’ resources are shared to improve ASP implementation in hospitals. 

The lack of and/or incompatibility of information technology (IT) systems with antimicrobial stewardship interventions hinders ASP implementation [15,25]. Innovation and integration of compatible IT systems improves the processes and outcomes of ASPs in hospitals [26]. Moreover, physicians’ resistance to ASP restrictions on prescribing antimicrobials also affects ASP implementation. In their study, Perozziello et al. [27] suggests that ASP education interventions, instead of restrictive ones, can improve physicians’ engagement with ASP implementation. In our study, education, early engagement with physicians, and trialing interventions prior to full implementation increased physicians’ engagement despite the initial resistance. This was also shown by Alawi et al. 2016 [28].

ASP implementation in the hospital reduced antimicrobial consumption and rates of some resistant strains, which further strengthens the evidence base for their effectiveness [28,29,30]. However, restricting antimicrobials may reduce resistance of some strains but increase the resistance of other strains, also known as the “‘squeeze the balloon effect” [31]. Resistance rates are also affected by the duration of antimicrobial treatment [32]. These should be considered when evaluating ASP outcomes. It is also important that hospitals monitor resistant rates, especially those of MDR, to target efforts to curb it [33]. Interesting perspectives are emerging, calling for a radical rethinking of what antimicrobial stewardship programs should entail, such as Vickers et al. calling for innovative commercial models to stimulate novel antimicrobial development, and integrating rapid diagnostics and infection control practices within the program [34]. Furthermore, given the enormity of the antimicrobial resistance threat, all possible strategies to identify novel or repurpose old agents to confer antimicrobial properties should be considered, including exploring the antimicrobial properties of essential oils [35].

The findings of our study can help Saudi hospitals develop and implement ASPs. We identified a number of challenges and the strategies to overcome them. Our findings, however, need to be interpreted with caution. First, our case study involves a single hospital. Although there are various lessons to be learnt on ASP implementation, there is no “one size fits all” approach, and other hospitals need to adapt the recommendations of our study before adopting this implementation model. Future research could use a comparative case study approach to analyze the similarities, differences and patterns across different hospitals. Second, we have not explored the effectiveness of ASP post implementation. Although we demonstrate that the ASP led to reduced antimicrobial consumption and a reduction in rates of resistance, an analysis of long-term effects is needed, through a longitudinal study, to understand if ASP processes and outcomes can be sustained. Third, our key informants included ASP team members and the hospital’s CEO. Exploring input from other ASP key players, such as hospital information technologists and middle managers could provide further insights on how interventions can be successfully implemented.

## 4. Methods

This study used a key stakeholder case study design [36], focusing on a tertiary care center (medical city) that implemented an antimicrobial stewardship program in 2016. The hospital has a 1500 bed capacity, consists of a coronary care unit (CCU), cardiac surgery intensive care unit (CSICU) and provides cardiac, hematology, oncology, neuroscience, medical and specialized surgery services. Qualitative methods were used, focusing on in-depth semi-structured interviews and analysis of relevant documents. The core members of the ASP team, including an ID consultant (Director of the ASP), a clinical pharmacist (Manager of the ASP), a consultant clinical microbiologist, an infection control consultant, and the CEO of the medical city were interviewed in July 2017, for 29–45 min. The interviews were conducted face-to-face in the participants’ main language (Arabic or English), audio recorded and transcribed verbatim. Additional data were collected through content analysis of 35 ASP hospital documents.

The interview schedule was developed following a review of the literature and discussions with three ASP pharmacists (two from Saudi Arabia and one from the UK) and two ID consultants (from Saudi Arabia). Questions in the schedule were all open-ended to obtain in-depth views and perspectives of the study participants. The interview schedule has two main sections. The first is a section on background information (three questions), such as the position of healthcare professionals, gender and years of experience. In the second section, 12 open-ended questions were used to explore the components of the ASP in the medical city, members of the ASP team and their responsibilities, the adoption and implementation process of the ASP, and the factors influencing the adoption and implementation process of the ASP in the medical city. Probing questions were also asked based on the responses of the participants to obtain further details.

## 5. Conclusions

Successful ASP implementation in Saudi hospitals is administrator-driven and requires a hospital leadership that empowers clinicians to take responsibility for implementing the program. Outsourcing expertise and resources could help hospitals address some of the implementation challenges. However, a compatible IT infrastructure that integrates ASP interventions is key to improving implementation and monitoring outcomes. 

## Figures and Tables

**Table 1:** Hospital antibiogram data (2015–2016) showing the percentage of sensitive susceptibilities between specific microorganisms (columns) and antibiotics (rows).

		Microorganisms					
Antibiotics	Year	*E. coli*	*A. baumannii*	*Enterobacter spp.*	*S. epidermidis*	*S. aureus*	*MRSA*
Amoxicillin/Clavulanic acid	2015	37	-	-	-	28	-
	2016	49	-	-	-	-	-
Cefepime	2015	45	28	39	-	-	-
	2016	48	22	66	-	-	-
Ciprofloxacin	2015	33	20	60	20	62	47
	2016	36	22	75	20	75	69
Clindamycin	2015	-	-	-		45	-
	2016	-	-	-	28	76	71
Colistin	2015	-	69	-	-	-	-
	2016	-	97	-	-	-	-
Gentamicin	2015	71	24	56	46	75	55
	2016	65	34	79	53	86	71
Piperacillin/Tazobactam	2015	47	-	48	-	-	-
	2016	77	-	64	-	-	-

**Table 2:** Hospital DOT data for July–Dec 2015 (before ASP implementation) and January–June 2016 (after).

Antibiotics	Total DOT (07–12/2015)	Total DOT (01–06/2016)
Tigecycline	1039.8	624.5
Colistin	2080.9	1417.2
Meropenem	2287.2	2597.3
Imipenem	2365.6	1666.1
TOTAL	7773.5	6305.1 (−18.9%)

## Data Availability

Data are contained within the article or Supplementary Material (Appendices 1 and 2).

## Supplementary Materials

The following are available online at https://www.mdpi.com/2079-6382/10/3/280/s1, Supplementary File S1: A copy of the hospital’s antimicrobial restriction form and the associated workflow is included; Supplementary File S2: Hospital antibiogram data (2015–2019) and DOT data (2018–2019) are included under Tables S1 and S2, respectively. Supplementary File S3: Hospital ASP updates.
