# Supplementary material for: Antimicrobial Stewardship Program Implementation in a Saudi Medical City: An Exploratory Case Study
Source: Antibiotics (Basel). 2021 Mar 9;10(3):280. doi: 10.3390/antibiotics10030280 (PMC8000012; doi:10.3390/antibiotics10030280)
Supplement: Supplementary file 1 [file antibiotics-10-00280-s001.zip › Appendices/Appendix A.docx]

|  | | | | PATIENT LABEL | |
| --- | --- | --- | --- | --- | --- |
| **ANTIMICROBIAL RESTRICTION FORM** | | | | | |
| Date: | | | | Unit: | |
| **FILLED BY PHYSICIAN** | | | | | |
| Ordering Physician:  Contact Number: | | | | ID Number:  Signature (or stamp): | |
| Culture withdrawn BEFORE antimicrobials therapy:  □ Yes □ No Date:  Culture withdrawn AFTER antimicrobials therapy:  □ Yes □ No Date: Restricted Antimicrobial  □ Teicoplanin □ Voriconazole  □ Linezolid □ Posaconazole  □ Tigecycline □ Caspofungin  □ Meropenem □ Micafungin  □ Imipenem □ Anidulafungin  □ Amikacin □ Amphotericin B  □ Colistin  Date Hospital of Admission: | | | | Indication (please specify infection SOURCE)  Justify not using narrower spectrum of activity  Prescribing Rationale  □ Pathogen-directed therapy  □ Empiric therapy  □ Prophylaxis  Estimated Duration of Therapy:  **Contact ID for approval “0515165026 ”**  ID Physician Approval Status  □ Approved □ ID was not contacted yet | |
| **FILLED BY PHARMACIST** | | | | | |
| Pharmacist: | | | | | |
| Dose | | | | Concurrent Antimicrobials (other than restricted):  Pharmacist Comments | |
| Culture  Source | Date | Organism | Comment |  |  |
|  |  |  |  |  |  |
|  |  |  |  |  |  |
|  |  |  |  |  |  |
|  |  |  |  |  |  |
| Labs  Tmax within 48 hours: UA:  WBC:  Neutrophil: ProCT:  CXR: | | | |  |  |
| **FILLED BY INFECTIOUS DISEASE PHYSICIAN** | | | | | |
| ID Physician: | | | | | |
| ID Physician Recommendations and Rationale | | | | | |
| □ Approved | | | | | □ Not Approved |
| Re-evaluation of approval for the restricted antimicrobial after ………. Days  Comments: | | | | | |

**Figure x.** Sample Antimicrobial Restriction Form

**Ordering physician**

- Fill out the antimicrobial restriction form
- Deliver the form to pharmacy ASAP
- Order restricted antibiotic for the estimated duration of therapy
- Contact ID for approval

**Assigned pharmacist**

- Review and complete the form no later than 18 hours after the pharmacy has received the form
- Deliver the form to the ID consultant
- ID will review forms by 9:30 am and 2 pm

**ID specialist**

- Review the form/order and reach a decision before order expiration
- Contact the ordering physician to discuss the case *(if ID was not contacted before)*
- If no response from the ordering physician, ID will contact the pharmacy to follow up with the ordering physician
- ID can decide if a formal consultation is required for approval

**Assigned Pharmacist**

- If ID approved, make sure that the duration of order matched the duration of therapy approved by ID.
- If ID did not approve the restricted antibiotic after 24 hours during weekdays and after 72 hours if ordered on Thursday after 12 pm, hold restricted antimicrobial agent and inform physician
- If ID is not available, continue the order until ID can review it.
- Re-assess the patient daily for dose adjustment, de-escalation or adverse drug reaction
- Document recommendation in the patient file, if applicable
- Sign off on the patient whenever the restricted antimicrobial is discontinued

**Figure y.** Antimicrobial Restriction Workflow
