# Supplementary material for: Antimicrobial Stewardship Program Implementation in a Saudi Medical City: An Exploratory Case Study
Source: Antibiotics (Basel). 2021 Mar 9;10(3):280. doi: 10.3390/antibiotics10030280 (PMC8000012; doi:10.3390/antibiotics10030280)
Supplement: Supplementary file 1 [file antibiotics-10-00280-s001.zip › Appendices/Appendix B.docx]

**Table 1s.** Tabular representation of the hospital antibiogram data (2015-2019). ASP was implemented in 2016. The values in the cells represent the percentage of sensitive susceptibilities between specific microorganisms (columns) and antibiotics (rows)

|  | Microorganisms | | | | | | | | | | |
| --- | --- | --- | --- | --- | --- | --- | --- | --- | --- | --- | --- |
| Antibiotics | **Year** | ***E. coli*** | ***K. pneumoniae*** | ***P. aeruginosa*** | ***A. baumannii*** | ***Enterobacter spp.*** | ***S. epidermidis*** | ***S. aureus*** | ***E. faecalis*** | ***E. faecium*** | ***MRSA*** |
| Amikacin | 2015 | 97 | 82 | 84 | 32 | 85 | - | - | - | - | - |
|  | 2016* | 90 | 74 | 85 | - | 92 | - | - | - | - | - |
|  | 2017 | 91 | 68 | 84 | - | 92 | - | - | - | - | - |
|  | 2018 | 97 | 69 | 78 | - | - | - | - | - | - | - |
|  | 2019 | 99 | 76 | 87 | - | - | - | - | - | - | - |
| Amoxicillin / Clavulanic acid | 2015 | 37 | 35 | - | - | - | - | 28 | - | - | - |
|  | 2016* | 49 | 42 | - | - | - | - | - | - | - | - |
|  | 2017 | 53 | 45 | - | - | - | - | - | - | - | - |
|  | 2018 | 57 | 44 | - | - | - | - | - | - | - | - |
|  | 2019 | 66 | 52 | - | - | - | - | - | - | - | - |
| Cefepime | 2015 | 45 | 45 | 67 | 28 | 39 | - | - | - | - | - |
|  | 2016* | 48 | 45 | 71 | 22 | 66 | - | - | - | - | - |
|  | 2017 | 42 | 36 | 63 | 22 | 80 | - | - | - | - | - |
|  | 2018 | 41 | 38 | 62 | 33 | - | - | - | - | - | - |
|  | 2019 | 40 | 51 | 72 | 69 | - | - | - | - | - | - |
| Ceftazidime | 2015 | - | - | 65 | 20 | - | - | - | - | - | - |
|  | 2016* | - | - | 58 | 19 | - | - | - | - | - | - |
|  | 2017 | - | - | 57 | 17 | - | - | - | - | - | - |
|  | 2018 | - | - | 59 | 32 | - | - | - | - | - | - |
|  | 2019 | - | - | 71 | 53 | - | - | - | - | - | - |
| Cefuroxime | 2015 | 34 | 30 | - | - | - | - | - | - | - | - |
|  | 2016* | 23 | 22 | - | - | - | - | - | - | - | - |
|  | 2017 | 32 | 23 | - | - | - | - | - | - | - | - |
|  | 2018 | 34 | 33 | - | - | - | - | - | - | - | - |
|  | 2019 | 36 | 44 | - | - | - | - | - | - | - | - |
| Ciprofloxacin | 2015 | 33 | 45 | 71 | 20 | 60 | 20 | 62 | - | - | 47 |
|  | 2016* | 36 | 55 | 67 | 22 | 75 | 20 | 75 | - | - | 69 |
|  | 2017 | 35 | 44 | 64 | 14 | 84 | 24 | 95 | - | - | 64 |
|  | 2018 | 37 | 44 | 70 | 27 | - | 53 | 74 | - | - | 60 |
|  | 2019 | 36 | 55 | 83 | 59 | - | 35 | 67 | 50 | - | - |
| Clindamycin | 2015 | - | - | - | - | - |  | 45) | - | - | - |
|  | 2016* | - | - | - | - | - | 28 | 76 | - | - | 71 |
|  | 2017 | - | - | - | - | - | 39 | 87 | - | - | 78 |
|  | 2018 | - | - | - | - | - | 55 | 82 | - | - | 70 |
|  | 2019 | - | - | - | - | - | 45 | 83 | - | - | - |
| Colistin | 2015 | - | - | - | 69 | - | - | - | - | - | - |
|  | 2016* | - | - | - | 97 | - | - | - | - | - | - |
|  | 2017 | - | - | - | 85 | - | - | - | - | - | - |
|  | 2018 | - | - | - | 81 | - | - | - | - | - | - |
|  | 2019 | - | - | - |  | - | - | - | - | - | - |
| Gentamicin | 2015 | 71 | 63 | 72 | 24 | 56 | 46 | 75 | - | - | 55 |
|  | 2016* | 65 | 65 | 71 | 34 | 79 | 53 | 86 | - | - | 71 |
|  | 2017 | 76 | 61 | 73 | 36 | 81 | 48 | 82 | - | - | 68 |
|  | 2018 | 78 | 62 | 69 | 43 | - | 69 | 85 | - | - | 60 |
|  | 2019 | 77 | 64 | 77 | - | - | 57 | 83 | - | - | - |
| Imipenem | 2015 | 100 | 77 | - | 2 | 89 | - | - | - | - | - |
|  | 2016* | 99 | 75 | 69 | 1 | 91 | - | - | - | - | - |
|  | 2017 | 96 | 65 | 64 | 5 | 94 | - | - | - | - | - |
|  | 2018 | 98 | 66 | 54 | 35 | - | - | - | - | - | - |
|  | 2019 | 99 | 75 | 65 | - | - | - | - | - | - | - |
| Linezolid | 2015 | - | - | - | - | - | - | - | 100 | 100 | - |
|  | 2016* | - | - | - | - | - | - | - | 100 | 98 | - |
|  | 2017 | - | - | - | - | - | - | - | 100 | 100 | - |
|  | 2018 | - | - | - | - | - | - | - | - | 100 | - |
|  | 2019 | - | - | - | - | - | - | - | 100 | - | - |
| Meropenem | 2015 | 100 | 78 | 72 | 7 | 88 | - | - | - | - | - |
|  | 2016* | 99 | 75 | 71 | 4 | 91 | - | - | - | - | - |
|  | 2017 | 96 | 65 | 64 | 6 | 94 | - | - | - | - | - |
|  | 2018 | 98 | 66 | 54 | 35 | - | - | - | - | - | - |
|  | 2019 | 99 | 74 | 66 | - | - | - | - | - | - | - |
| Norfloxacin | 2015 | 40 | 36 | - | - | - | - | - | - | - | - |
|  | 2016* | 34 | 44 | - | - | - | - | - | - | - | - |
|  | 2017 | 46 | 48 | - | - | - | - | - | - | - | - |
|  | 2018 | 30 | 56 | - | - | - | - | - | - | - | - |
|  | 2019 | - | - | - | - | - | - | - | - | - | - |
| Piperacillin / Tazobactam | 2015 | 47 | 46 | 73 | - | 48 | - | - | - | - | - |
|  | 2016* | 77 | 57 | 54 | - | 64 | - | - | - | - | - |
|  | 2017 | 78 | 52 | 35 | - | 63 | - | - | - | - | - |
|  | 2018 | 78 | 55 | 55 | - | - | - | - | - | - | - |
|  | 2019 | 86 | 64 | 69 | - | - | - | - | - | - | - |
| Vancomycin | 2015 |  | - | - | - | - | 100 | 100 | 95 | 55 | 100 |
|  | 2016* | - | - | - | - | - | 100 | 100 | 100 | 48 | 100 |
|  | 2017 | - | - | - | - | - | 100 | 100 | 95 | 53 | 100 |
|  | 2018 | - | - | - | - | - | 100 | 100 | 99 | - | 100 |
|  | 2019 | - | - | - | - | - | 100 | 100 | 98 | - | - |

**Table 2s.** Hospital Days of Therapy (DOT) data for 2018 and 2019

| Total DOT per 1000 patients' days | | | |  |  |
| --- | --- | --- | --- | --- | --- |
| **Antimicrobials** | | **2018** | **2019** |  | |
| Tigecycline | | 2720 | 1069 |  | |
| Colistin IV | | 3876 | 2596 |  | |
| Meropenem | | 7890 | 16596 |  | |
| Imipenem | | 3709 | 4969 |  | |
| Amikacin | | 337 | 264 |  | |
| Linezolid IV | | 91 | 345 |  | |
| Voriconazole IV | | 120 | 445 |  | |
| Amphotericin | | 200 | 888 |  | |
| Micafungin | | 654 | 2154 |  | |
| **TOTAL** | | 19597 | 29362 |  | |
