# Supplementary material for: Antimicrobial Stewardship Program Implementation in a Saudi Medical City: An Exploratory Case Study
Source: Antibiotics (Basel). 2021 Mar 9;10(3):280. doi: 10.3390/antibiotics10030280 (PMC8000012; doi:10.3390/antibiotics10030280)
Supplement: Supplementary file 1 [file antibiotics-10-00280-s001.zip › Appendices/Appendix C.docx]

**Hospital ASP updates (2020-2021)**

*Status of ASP (2020-2021)*

The ASP committee reported that the ASP is a more developed and mature programme. The ASP committee is still meeting regularly, reporting the ASP KPIs directly to the hospital CEO and CMO (Chief Marketing Officer). The committe collaborates closely and maintains frequent communication with the Pharmacy & Therapeutics committee and the Infection Control committee.

The hospital has now implemented a new information system (since May 2019). The new system is compatible with the ASP programme requirements especially in relation to the prescribing/ dispensing of antimicrobials requiring pre-authorisation. The new system also generates automatic DOT reports and floor-specific and MDRO-specific antibiograms (these have been generated manually prior to the implementation of the new system). As a result, ASP processes are “more organized and the results are real”. The ASP committee’ s latest efforts include extending the list of restricted antimicrobials with a close montoring of the rates of surgical site infections and surgical prophalxis antibiotics prescribing.

*Antimicrobial resistance rates*

Since implementing the ASP, the hospital achieved a reduction in the rates of some antimicrobial resistant strains, particularly those of *Acinetobacter baumannii*. This is reduction has been sustained. However, there has been an increase in rates of CRE (cephalosporin-resistant Enterobacteriaceae) and ESBL isolates. Various recommendations and taskforces are in place to address this issue particularly antimicrobials prescribing for central-line associated bloodstream infections and surgical site infections

*Appropriateness of antimicrobials’ prescribing*

Prescribing restriced antimicrobials is no longer problematic; there is great adherence to the process, and all pre-authorisation forms still require ID approval. However, adherence to the recommended duration of antimicrobial therapy (following ID approval) remains an issue. In addition, the inappropriate use of non-restricted antimicrobials remains high, particularly for surgical prophylaxis and in the outpatient setting.

*ASP Successes (4 years since implementation)*

- Reduction in number of resistant *Acinetobacter baumannii* isolates (from 90s to 30s)
- Increased sensitivity to aminoglycosides, piperacillin/tazobactam, carbapenems and colistin
- Reduced DOT of tigecycline
- Setting up OPAT (Outpatient Parenteral Antimicrobial Therapy) services
- Implementation of antimicrobial prescribing guidelines (e.g. vancomycin and surgical prophylaxis)

*ASP Challenges (4 years since implementation)*

- Low adherence to surgical prophylaxis guidelines
- Antifungal antibiogram still not implemented
- De-escalation of non-restricted antibiotics
